# Seasonality of mortality under a changing climate: a time-series analysis of mortality in Japan between 1972 and 2015

**DOI:** 10.1186/s12199-021-00992-8

**Published:** 2021-07-03

**Authors:** Lina Madaniyazi, Yeonseung Chung, Yoonhee Kim, Aurelio Tobias, Chris Fook Sheng Ng, Xerxes Seposo, Yuming Guo, Yasushi Honda, Antonio Gasparrini, Ben Armstrong, Masahiro Hashizume

**Affiliations:** 1 Department of Pediatric Infectious Diseases, Institute of Tropical Medicine, Nagasaki University, Nagasaki, Japan; 2 School of Tropical Medicine and Global Health, Nagasaki University, Nagasaki, Japan; 3 Department of Mathematical Sciences, Korea Advanced Institute of Science and Technology, Daejeon, South Korea; 4 Department of Global Environmental Health, Graduate School of Medicine, The University of Tokyo, Tokyo, Japan; 5 Institute of Environmental Assessment and Water Research (IDAEA), Spanish Council for Scientific Research (CSIC), Barcelona, Spain; 6 Department of Epidemiology and Preventive Medicine, School of Public Health and Preventive Medicine, Monash University, Melbourne, Australia; 7 Climate, Air Quality Research Unit, School of Public Health and Preventive Medicine, Monash University, Melbourne, Australia; 8 Faculty of Health and Sport Sciences, University of Tsukuba, Tsukuba, Japan; 9 Department of Public Health, Environments and Society, London School of Hygiene & Tropical Medicine, London, UK; 10 Centre on Climate Change and Planetary Health, London School of Hygiene & Tropical Medicine, London, UK; 11 Centre for Statistical Methodology, London School of Hygiene & Tropical Medicine, London, UK; 12 Department of Global Health Policy, Graduate School of Medicine, The University of Tokyo, 7-3-1, Hongo, Bunkyo-ku, Tokyo, Japan

**Keywords:** Seasonality, Mortality, Temperature, Climate change

## Abstract

**Background:**

Ambient temperature may contribute to seasonality of mortality; in particular, a warming climate is likely to influence the seasonality of mortality. However, few studies have investigated seasonality of mortality under a warming climate.

**Methods:**

Daily mean temperature, daily counts for all-cause, circulatory, and respiratory mortality, and annual data on prefecture-specific characteristics were collected for 47 prefectures in Japan between 1972 and 2015. A quasi-Poisson regression model was used to assess the seasonal variation of mortality with a focus on its amplitude, which was quantified as the ratio of mortality estimates between the peak and trough days (peak-to-trough ratio (PTR)). We quantified the contribution of temperature to seasonality by comparing PTR before and after temperature adjustment. Associations between annual mean temperature and annual estimates of the temperature-unadjusted PTR were examined using multilevel multivariate meta-regression models controlling for prefecture-specific characteristics.

**Results:**

The temperature-unadjusted PTRs for all-cause, circulatory, and respiratory mortality were 1.28 (95% confidence interval (CI): 1.27–1.30), 1.53 (95% CI: 1.50–1.55), and 1.46 (95% CI: 1.44–1.48), respectively; adjusting for temperature reduced these PTRs to 1.08 (95% CI: 1.08–1.10), 1.10 (95% CI: 1.08–1.11), and 1.35 (95% CI: 1.32–1.39), respectively. During the period of rising temperature (1.3 °C on average), decreases in the temperature-unadjusted PTRs were observed for all mortality causes except circulatory mortality. For each 1 °C increase in annual mean temperature, the temperature-unadjusted PTR for all-cause, circulatory, and respiratory mortality decreased by 0.98% (95% CI: 0.54–1.42), 1.39% (95% CI: 0.82–1.97), and 0.13% (95% CI: − 1.24 to 1.48), respectively.

**Conclusion:**

Seasonality of mortality is driven partly by temperature, and its amplitude may be decreasing under a warming climate.

**Supplementary Information:**

The online version contains supplementary material available at 10.1186/s12199-021-00992-8.

## Introduction

Global temperatures have been increasing since the pre-industrial era. In the Northern Hemisphere, the period 1983–2012 may have been the warmest of the past 1400 years [1]. Without any mitigation, global surface temperature is projected to increase by 2.6–4.8 °C on average by 2081–2100 over that of 1986–2005 [1]. Over time, the amplitude of the annual temperature cycle has decreased, and winter is warming faster than summer [2]. These trends in warming and reduced amplitude of the annual temperature cycle pose a serious threat to organisms and their ecosystems. The phenomenon has also resulted in shifting seasonal behaviors of various species [3].

Human health exhibits a seasonal pattern that has been documented since the time of Hippocrates [4]. Seasonality of mortality in particular has received much attention. Mortality generally follows a notable seasonal pattern with a predominant winter peak and a trough in late summer or early autumn in regions that have distinct seasonal weather conditions [5]. However, this was not always the case. The seasonality of all-cause mortality in Japan was reported to have changed from a summer peak in the 1920s to a winter peak in the 1960s, and that mortality may have been de-seasonalized in other countries such as the USA [6]. This pattern reflects the complex interactions between humans and the environment.

Ambient temperature (hereafter temperature) is a major environmental element long known to have contributed to this seasonality [7]. Exposure to cold or hot temperatures can lead to adverse health outcomes and mortality [8]. Therefore, a warming climate and lower amplitude of the annual temperature cycle may reduce the mortality risks of cold temperatures and probably increase those of hot temperatures, which may reduce the amplitude of mortality by season. However, few studies have examined this topic so far [9, 10]. Knowledge of how seasonality of mortality is affected by a changing climate will strengthen our understanding of the health impacts of climate change and provide important information for future management of healthcare demand across seasons.

Investigating the impact of a warming climate on seasonality of mortality requires the understanding of two issues. First, how much of the seasonality of mortality is directly related to temperature. To date, only a few studies have assessed the contribution of temperature to the seasonality [11, 12], and their analyses were typically based on monthly data and simplified methods that may fail to capture the non-linear and delayed short-term effects of daily temperature on mortality. A sophisticated statistical approach is required to improve the previous methods. Second, time-varying factors other than a warming climate (e.g., hygiene, lifestyle, medical treatment, and housing conditions) may also influence seasonality changes over time. These potential confounders should be considered when linking the warming climate to a changing seasonality of mortality, and the resulting numbers must be interpreted cautiously.

This study investigated the seasonality of mortality in the context of a warming climate, with a particular focus on its amplitude, by analyzing daily time-series data of mortality in 47 prefectures in Japan between 1972 and 2015. We first estimated and compared the amplitude of seasonal variation in mortality before and after temperature adjustment (i.e., removing the short-term effect of temperature on mortality) to address our first hypothesis that seasonality of mortality is substantially related to temperature. Next, we investigated temporal changes in the seasonal amplitude of mortality without temperature adjustment and its association with annual mean temperature controlling for other potential time-varying confounders to address our second hypothesis that a warming climate is likely to reduce the seasonal amplitude of mortality.

## Methods

### Data collection

Daily time-series data on mean temperature and mortality counts between 1972 and 2015 were collected for each prefecture, with the exception of Okinawa, where data were from 1973 to 2015. Daily mortality cases were obtained from the Ministry of Health, Labour and Welfare of Japan, including all-cause mortality, circulatory mortality (International Classification of Disease: ICD-8 codes 390–458, ICD-9 codes 390–459, and ICD-10 codes I00–I99), respiratory mortality (ICD-8 and ICD-9 codes 460–519 and ICD-10 codes J00–J99), and mortality from influenza (ICD-8 codes 470–474, ICD-9 codes 487–488, and ICD-10 codes J09–J11).

Annual mean temperature was calculated for each prefecture from 1972 to 2015 to investigate its relationship to the temporal changes in seasonality. To account for other potential confounders of the association between temperature and seasonality of mortality, annual data on prefecture-specific characteristics were collected, including relative humidity, demographics, macroeconomics, and prevalence of air conditioning in households with two or more inhabitants. For demographic factors, the proportion of individuals aged ≥65 years in each study year was considered in our investigation, because large seasonal fluctuations in mortality have been observed among the elderly in previous studies [13–15]. Economic development and housing conditions have also been linked to seasonality of mortality [16]; thus, we included the consumer price index from 1972 to 2012 and the prevalence of air conditioning from 1972 to 2009. Latitude and longitude data of the capital cities were also collected for each prefecture. The data collection for prefecture-specific characteristics has been described in detail previously [17].

### Statistical analysis

The statistical framework is summarized in Figure S1. In brief, in the first stage, we estimated seasonality without and with temperature adjustment by applying time-series regression models [8] to the data for each prefecture and a meta-analysis for pooling the prefecture-specific estimates. This is done by using the data for the overall study period of 44 years. In the second stage, we estimated seasonality without temperature adjustment for each year by using the annual data. Next, we evaluated the associations between annual mean temperature and temperature-unadjusted seasonality estimates in each year through a meta-analytical model. The analysis was conducted for all-cause, circulatory, and respiratory mortality separately.

### Seasonality attributable to temperature

We first applied time-series regression using generalized linear models with Poisson distribution accounting for overdispersion to estimate seasonality of mortality for each prefecture, using 44 years of data. Day of year was treated as an indicator for seasonality (from 1 to 366). A cyclic spline [19] with four degrees of freedom (*df*) was applied to the day of year to estimate the number of mortality cases on each day. The days with the maximum and minimum estimated mortality were identified as peaks and troughs, respectively. The peak-to-trough ratio (PTR) of mortality estimates was then estimated as a measure of seasonal amplitude (Figure S2). Long-term trends and effects of day of week were controlled using strata defined by year, day of week, and their interaction. Because of the potential effect of influenza on the seasonal pattern of mortality, mortality from influenza on the same day, used as a measure of circulating influenza, was natural log-transformed and adjusted using a natural cubic spline with three *df*. In addition, we added one to the observations in the time series of daily mortality from influenza, as there were days when the mortality from influenza was zero. We tested our choices on *df* for spline functions, and PTRs remained similar (Table S1).

Next, we introduced daily temperature into the model described above to remove the short-term effect of temperature on mortality and to obtain the temperature-adjusted PTR. A bi-dimensional cross-basis function [8] was used to account for the non-linear and lagged effects of temperature on mortality: a natural cubic B-spline basis with three internal knots at the 25th, 50th, and 75th percentiles of temperature distribution for each prefecture was used for exposure-response association, and another natural cubic B-spline basis with 3 *df* with extended lag up to 21 days was used for the lag-response association. We tested the modeling choices in a sensitivity analysis.

We pooled the prefecture-specific PTRs across all 47 prefectures, using a random-effects meta-analysis treating prefectures as a random effect. PTRs before and after the temperature adjustments were compared to assess the contribution of temperature to the seasonality of mortality (hereafter, PTR refers to temperature-unadjusted PTR, unless specified otherwise). More details were provided in the supplementary material on the seasonality assessment.

### Temporal changes in the seasonal amplitude and their association with warming temperature

We applied the generalized linear models described above to assess seasonality in each prefecture for each year and to obtain the prefecture-specific temperature-unadjusted PTR for each year. Multilevel multivariate meta-regression models [18] were then used to investigate the relationships between annual mean temperature and annual PTR. Prefecture-specific seasonality estimates for each year (with natural logarithm transformation of PTR) were the outcome. Prefectures and year nested within prefectures were treated as random terms. Time trend was modeled using a natural cubic spline with two *df* for year. The latitude and longitude of each prefecture were also included to account for spatial correlations.

In our initial investigation, the relationship of PTR and annual mean temperature or annual data on other prefecture-specific characteristics (including relative humidity, proportion of population aged ≥65 years, consumer price index, and prevalence of air conditioning) was examined individually in a separate model. Next, the association between annual mean temperature and PTR was estimated by controlling for all other prefecture-specific characteristics as a priori covariates.

All statistical analyses were conducted in R (version 3.6.1) using the packages *dlnm* and *mixmeta*.

## Results

Monthly mean temperature ranged from 4.17 °C in January to 26.72 °C in August with an average of 15.12 °C for the entire study period (Table 1), and an increasing trend was observed between 1972 and 2015 (Figure S4). A significant seasonal pattern was observed for mortality, with the highest number of cases in January and the lowest number of cases in September (Figures S3 and S4).

**Table 1:** Monthly summary of daily mean temperature and daily mortality cases averaged over 47 prefectures between 1972 and 2014 (mean ± standard deviation)

Month	Mean temperature (°C)	All-cause mortality (cases)	Circulatory mortality (cases)	Respiratory mortality (cases)
January	4.17 ±3.99	61.88 ±55.01	22.92 ±17.48	8.55 ±8.93
February	4.75 ±4.31	59.98 ±49.11	21.84 ±16.49	8.49 ±8.39
March	7.91 ±4.29	56.91 ±66.01	20.2 ±15.17	7.58±7.44
April	13.36 ±4.01	53.1 ±43.63	18.36 ±13.9	6.8 ±6.93
May	18 ±3.25	50.23 ±41.53	16.85 ±12.91	6.27 ±6.54
June	21.74 ±2.9	47.66 ±39.94	15.42 ±12.09	5.75 ±6.06
July	25.54 ±3.05	47.86 ±40.59	15.18 ±12.22	5.71 ±6.03
August	26.72 ±2.54	48.05 ±40.92	15.03 ±12.12	5.75 ±6.14
September	22.98 ±3.31	47.58 ±40.33	14.94 ±11.78	5.56 ±6.09
October	17.33 ±3.61	50.58 ±42.29	16.75 ±12.91	5.9 ±6.38
November	11.7 ±4.18	53.98 ±45.01	18.7 ±14.35	6.5 ±6.96
December	6.58 ±4.07	57.41 ±47.78	20.67 ±15.84	7.15 ±7.49
Whole year	15.12 ±8.61	52.91 ±46.88	18.05 ±14.31	6.66 ±7.08

**Fig. 1:**
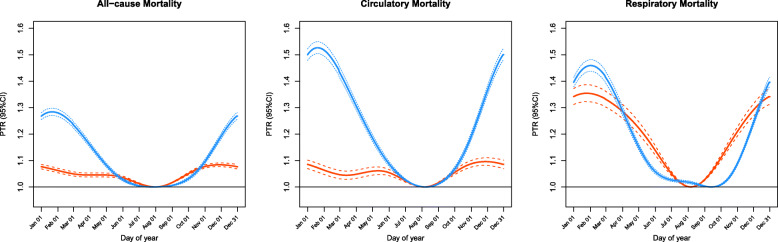
Seasonality of mortality for Japan as a whole, obtained by pooling 47 prefecture-specific estimates without (blue) and with (red) temperature adjustment. The seasonality, here referring to the association between the day-of-year and mortality, is computed as the relative risk (RR) of mortality estimates at each day to the minimum mortality estimate at the trough with 95% confidence intervals (95%CIs): $\mathrm{RR}=\frac{\mathrm{Mortality}\ \mathrm{estimate}\ \mathrm{at}\ {\mathrm{day}}_{\mathrm{i}}}{\mathrm{Minimum}\ \mathrm{mortality}\ \mathrm{estimate}\ \mathrm{at}\ \mathrm{the}\ \mathrm{trough}}$

Figure 1 shows the pooled estimated seasonal pattern of mortality for Japan as a whole. Prior to temperature adjustment, we observed a marked seasonal pattern with low mortality estimates in summer/autumn and high mortality estimates in winter. The pooled PTRs for all-cause, circulatory, and respiratory mortality were 1.28 (95% confidence interval (CI): 1.27–1.30), 1.53 (95% CI: 1.50–1.55), and 1.46 (95% CI: 1.44–1.48), respectively. After adjusting for temperature, the shape of seasonality was similar, but the amplitudes were lower (Fig. 1). The pooled temperature-adjusted PTRs for all-cause, circulatory, and respiratory mortality were 1.08 (95% CI: 1.08–1.10), 1.10 (95% CI: 1.08–1.11), and 1.35 (95% CI: 1.32–1.39), respectively. Circulatory mortality showed a larger reduction in PTR after temperature adjustment than respiratory mortality. Prefecture-specific assessments showed similar results (Table S2).

**Fig. 2:**
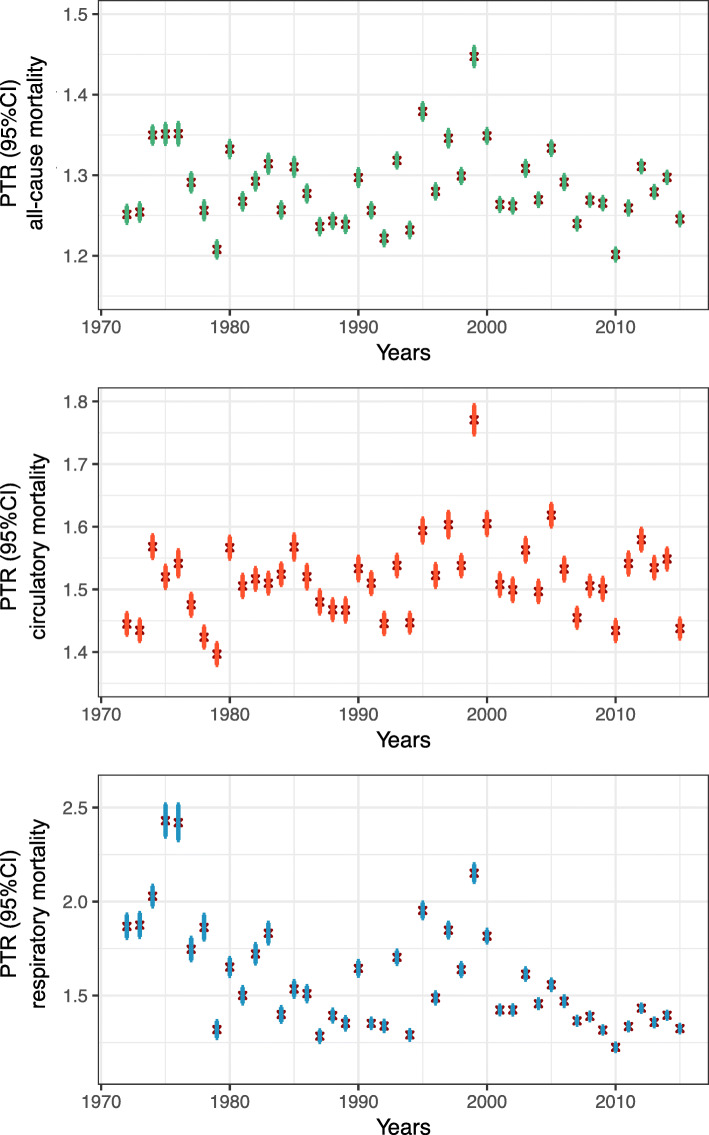
Annual peak-to-trough ratios (PTR) with 95% confidence intervals (95% CI) for Japan as a whole, without temperature adjustment for all-cause (green), circulatory (red), and respiratory mortality (blue)

**Fig. 3:**
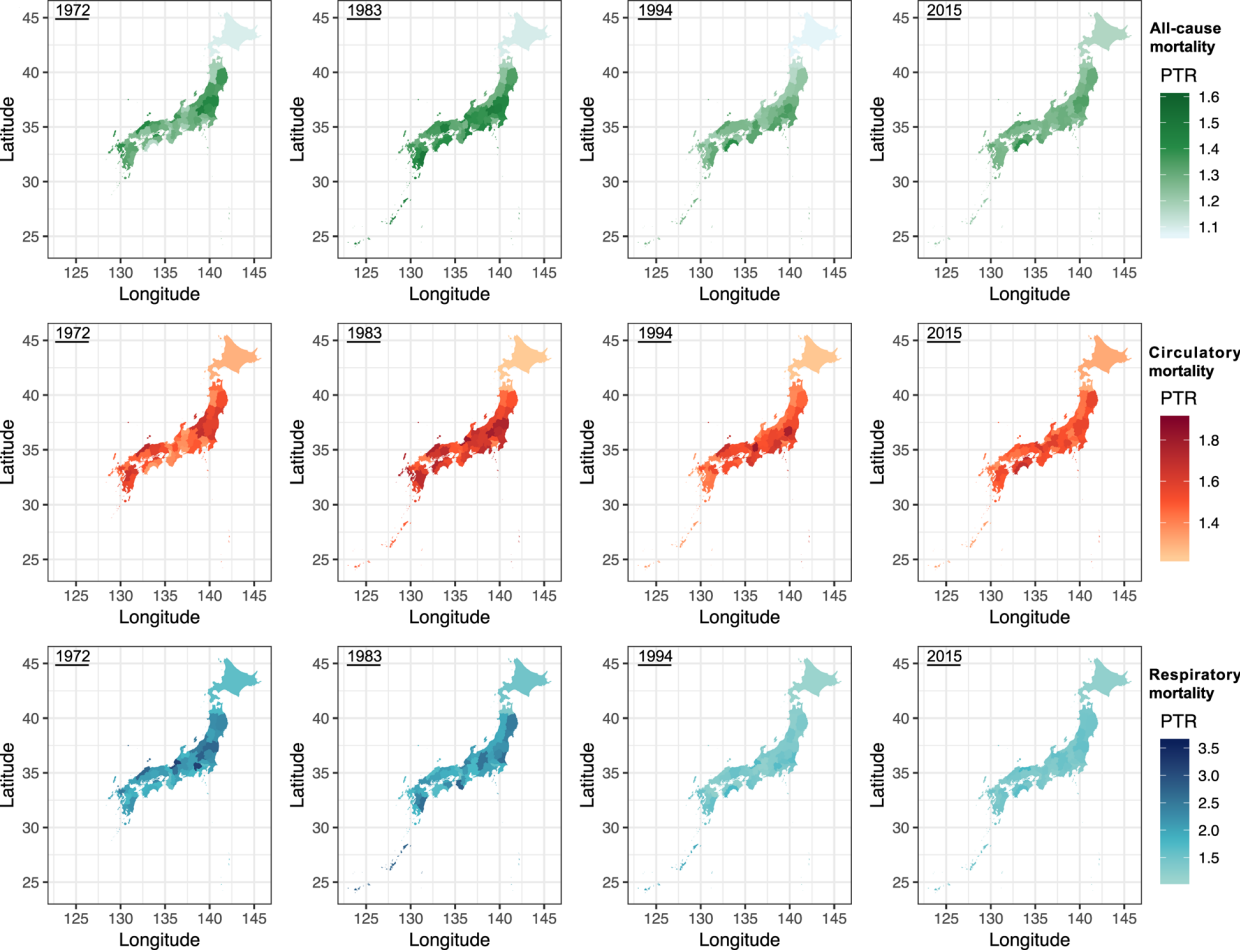
Prefecture-specific peak-to-trough ratios (PTR) without temperature adjustment in 1972, 1983, 1994, and 2015 for all-cause (green), circulatory (red), and respiratory mortality (blue)

A slight decreasing trend was observed for PTRs for all-cause mortality, and a significant decreasing trend was observed for respiratory mortality, but no obvious time trend was observed for circulatory mortality (Fig. 2). Prefecture-specific PTRs in 1972, 1983, 1994, and 2015 (Fig. 3) for all-cause and respiratory mortality exhibited an overall reduction, with the largest reduction observed in eastern and southern prefectures. There was no significant change in PTRs for circulatory mortality.

Figure S7 shows the temporal changes in each meta-predictor during the study period. In general, a decreasing trend was observed for relative humidity and an increasing trend was observed for temperature, proportion of individuals aged ≥65 years, consumer price index, and the prevalence of air conditioning. In particular, annual mean temperature increased from 14.6 °C in 1972 to 15.9 °C in 2015 (Figure S7). Prior to adjusting for other covariates, annual mean temperature was negatively associated with PTR (Table 2). Each 1 °C increase in annual mean temperature reduced the PTR for all-cause, circulatory, and respiratory mortality by 1.62% (95% CI: 1.19–2.10), 2.07% (95% CI: 1.53–2.70), and 2.25% (95% CI: 1.00–3.49), respectively. After adjusting for covariates, negative associations between annual mean temperature and PTR remained: each 1 °C increase in annual mean temperature reduced the PTR for all-cause, circulatory, and respiratory mortality by 0.98% (95% CI: 0.54–1.42), 1.39% (95% CI: 0.82–1.97), and 0.13% (95% CI: − 1.24 to 1.48), respectively. The relationship of each covariate with the PTR from the meta-regression models with and without adjusting for other covariates is shown in Tables S3 and S4, respectively.

**Table 2:** Relationship between annual mean temperature (°C) and seasonality estimates^*^, expressed as the percent changes in temperature-unadjusted peak-to-trough ratio (95% confidence interval) for 1 °C increase in annual mean temperature

Models	All-cause mortality	Circulatory mortality	Respiratory mortality
Unadjusted for confounders	− 1.62 (− 2.10 to − 1.19)	− 2.07 (− 2.70 to − 1.53)	− 2.25 (− 3.49 to − 1.00)
Adjusting for confounders^§^	− 0.98 (− 1.42 to − 0.54)	− 1.39 (− 1.97 to − 0.82)	− 0.13 (− 1.4 to 1.24)

^§^Confounders include relative humidity, proportion of population aged ≥ 65 years, consumer price index, and prevalence of air conditioning

^*^Meta-regression models were used to investigate the relationships between annual mean temperature and seasonality estimates. Temperature-unadjusted seasonality estimates for each year in each prefecture (with natural logarithm transformation of temperature-unadjusted PTR) were the outcome. The percent changes in temperature-unadjusted PTR were calculated as 100(exp(β)-1), where β is the regression coefficient for log (PTR) on annual temperature (°C)

## Discussion

In this study, we investigated the seasonality of mortality under a warming climate in Japan. We found that PTRs in all-cause, circulatory, and respiratory mortality for Japan as a whole decreased after adjusting for temperature. Furthermore, seasonality tended to be flattened for respiratory and all-cause mortality between 1972 and 2015. The changing seasonal amplitude was negatively associated with annual mean temperature. These negative associations remained significant after adjusting for other time-varying prefectural characteristics for all-cause and circulatory mortality, but the association with respiratory mortality tended toward to null. Although confirmation is required, our results suggest that a warming climate may lead to a flattening of seasonality when other potential confounders remain similar over time.

This seasonal pattern of mortality in Japan is consistent with findings from other studies conducted in regions with four distinct seasons [7, 9, 13, 15, 20, 21]. Similar seasonal patterns were reported for other health indicators, such as levels of C-reactive protein, a marker of inflammation [22]. Various seasonal factors have been proposed to explain these patterns, including temperature, exposure to sunlight, human activity patterns, and the incidence of influenza [7, 12, 15]. Of these, temperature has gained the most attention from researchers. Gasparrini and colleagues [8] collected data on temperature and mortality in over 600 cities worldwide and observed that increased mortality was associated with both hot and cold temperatures. Relationships between temperature and key cardiac risk factors have also been reported [23]. Cold temperatures can lead to peripheral vasoconstriction to reduce thermal conduction, increase metabolic heat production, trigger platelet activation, and provoke cumulative increases in inflammatory markers [7]. Conversely, heat stress can lead to generalized peripheral vasodilatation and over-sweating, provoking increases in heart rate and hyperthermia and even syncope and myocardial ischemia [7].

We found that the amplitude of seasonality was reduced after controlling for temperature. However, the amplitude reduction varied between all-cause, circulatory, and respiratory mortality, with the largest reduction observed for circulatory mortality and the smallest for respiratory mortality. Using aggregated monthly data on mortality at the national level from 1970 to 1999, Nakaji and colleagues [12] identified a similar seasonal pattern and reduced seasonal amplitude when monthly temperature was considered in the analysis, despite having used a linear function for temperature. Furthermore, consistent with our findings, no notable reduction was observed for respiratory mortality after adjusting for temperature in that study [12]. Our findings suggest that temperature is potentially a crucial contributor to seasonal variations in mortality, and especially to circulatory mortality.

The reduced seasonal amplitudes observed after adjusting for temperature in our analysis, coupled with the existing extensive evidence of temperature effects on mortality, led us to hypothesize that the amplitude of seasonality may be undergoing reduction as temperatures increase under conditions of climate change. Over the study period, annual mean temperature in Japan increased from 14.6 °C in 1972 to 15.9 °C in 2015, while PTRs decreased for all-cause and respiratory mortality. We found that annual mean temperature was negatively related to PTR for all-cause, respiratory, and circulatory mortality. This association persisted for all-cause and circulatory mortality even after adjusting for potential confounders. Warmer winters and fewer cold periods may result in lower mortality peaks in winter, whereas warmer summers and more frequent and intense heat waves may lead to a rise in mortality in summer, thereby flattening the seasonality of mortality over time. In addition, fewer cold-related deaths in warmer winters may also lead to a shift in the population susceptible to warmer summers and may result in more deaths in the warm seasons, translating to a flatter seasonality [24, 25]. Therefore, the flattening of seasonality in Japan may be related to a warming climate. A recent global projection [26] of temperature-related mortality predicted a decrease in cold-related mortality and an increase in heat-related mortality in East Asia including Japan, without considering potential changes in demographics and adaptation. A warming climate could produce a flatter seasonality of mortality in Japan in the future, unless confounded by other factors.

It is important to emphasize that our findings should be interpreted cautiously. Other than the warming climate, we also found that changing seasonal amplitude was associated with lower relative humidity, population aging, economic development, and increasing prevalence of air conditioning. Populations are likely to adapt to a changing climate. Heat-related mortality in the warm seasons may be reduced, and seasonal amplitude could remain consistent over time. In addition, efficient protection measures against seasonal risks, such as vaccination against infectious diseases, may prevent excess winter mortality, also in turn reducing the seasonal amplitude.

To our knowledge, so far only two studies [9, 10] have investigated the relationship between warming climate and changes in the seasonality of mortality, and the conclusions of these studies are similar to ours. Bennett and colleagues [10] reported that the ratio of summer-to-winter deaths in those aged 55 years and above in Australia increased from 0.71 to 0.86 between 1968 and 2007 in tandem with rising annual temperatures. McGregor and colleagues [9] found a decrease in seasonal amplitude for mortality from ischemic heart disease between 1974 and 1999 in five English counties, and this trend was positively correlated with the amplitude of the annual temperature cycle. However, these two studies relied on a relatively simple method of using monthly aggregated data to assess seasonality, and their results are limited to a specific population, region, or cause of mortality; moreover, they did not include recent years. We included recent time-varying annual data on prefecture-specific characteristics in the meta-regression analysis, which enabled us to gain a better understanding of the impacts of climate change on the seasonality of mortality by taking into account potential confounders.

Some limitations should be noted. First, we focused on the amplitude of seasonal variation in mortality and did not consider the changes in the shape of seasonal patterns (i.e., peak and trough). Although the shape of seasonal patterns did not appear to change substantially during our study period, it is possible that a warming climate may impact the timing of peak and/or trough. Second, climate change includes rising temperatures, shortening winter seasons, increasing extreme weather events, etc., but we only considered the increasing annual mean temperature in this study. Third, our investigation was conducted in Japan, where the seasons are distinct in most prefectures. Hence, future investigations in other locations with different climates are required to confirm our findings.

## Conclusion

In this study, we investigated the seasonality of mortality in Japan, and in particular, its response to a warming climate, and has several methodological strengths. First, we used daily rather than monthly mortality data covering 44 years in Japanese prefectures to assess seasonality. Second, we assessed the seasonality with and without adjusting for temperature, and this adjustment was conducted using a distributed lag non-linear structure. Third, time-varying data on prefecture-specific characteristics were continuously available for many years, enabling us to account for their potential impact on seasonality. One highlight of our findings is the negative relationship observed between annual mean temperature and the amplitudes of seasonality, suggesting a potential impact of climate change on seasonality of human health outcomes. Although further investigations are required to confirm our findings, this study adds important evidence to the existing profile of climate change-related health impacts that will contribute to the management of healthcare demands throughout the year under ongoing climate change.

## Supplementary Information

**Additional file 1:.** Figure S1. Flowchart illustrating the main stages of the statistical analysis. Statistical analysis for Stage I: Seasonality assessment. Table S1. The pooled peak-to-trough ratio (95% confidence intervals) for Japan as a whole for all-cause mortality by using different degrees of freedom (df) for cyclic spline* and natural cubic spline§. Table S2. The 44-year averaged prefecture-specific peak-to-trough ratios (95% confidence intervals) for all-cause, circulatory and respiratory mortality. Table S3. The relationship (slope estimate (95% confidence intervals)) between each prefecture-specific meta-predictor and PTR before adjusting for other meta-predictors. Table S4. The relationship (slope estimate (95% confidence intervals)) between each prefecture-specific meta-predictor and PTR after adjusting for all the other meta-predictors. Figure S3. Monthly mean of daily mean temperature and daily mortality cases at national level between 1972 and 2015. Figure S4. Daily mean temperature and daily mortality cases from 1972 to 2015 at national level. The spatial distribution of averaged daily mean temperature and mortality cases. Spatial distribution of PTR before and after temperature adjustment by using the data for the overall study period of 44 years. Figure S7. Time-series scatter plot for annual data on each prefecture-specific characteristic.

## Data Availability

Data are available upon reasonable request. The technical appendix, statistical code, and data set will be available upon request from the first author.
