# Supplementary material for: Seasonality of mortality under a changing climate: a time-series analysis of mortality in Japan between 1972 and 2015
Source: Environ Health Prev Med. 2021 Jul 3;26:69. doi: 10.1186/s12199-021-00992-8 (PMC8254906; doi:10.1186/s12199-021-00992-8)
Supplement: Supplementary file 1 — Additional file 1:. Figure S1. Flowchart illustrating the main stages of the statistical analysis. Statistical analysis for Stage I: Seasonality assessment. Table S1. The pooled peak-to-trough ratio (95% confidence intervals) for Japan as a whole for all-cause mortality by using different degrees of freedom (df) for cyclic spline* and natural cubic spline§. Table S2. The 44-year averaged prefecture-specific peak-to-trough ratios (95% confidence intervals) for all-cause, circulatory and respiratory mortality. Table S3. The relationship (slope estimate (95% confidence intervals)) between each prefecture-specific meta-predictor and PTR before adjusting for other meta-predictors. Table S4. The relationship (slope estimate (95% confidence intervals)) between each prefecture-specific meta-predictor and PTR after adjusting for all the other meta-predictors. Figure S3. Monthly mean of daily mean temperature and daily mortality cases at national level between 1972 and 2015. Figure S4. Daily mean temperature and daily mortality cases from 1972 to 2015 at national level. The spatial distribution of averaged daily mean temperature and mortality cases. Spatial distribution of PTR before and after temperature adjustment by using the data for the overall study period of 44 years. Figure S7. Time-series scatter plot for annual data on each prefecture-specific characteristic. [file 12199_2021_992_MOESM1_ESM.docx]

**Supplementary Material**

**Seasonality of mortality under a changing climate: a time-series analysis of mortality in Japan from 1972 to 2015**

Lina Madaniyazi, Yeonseung Chung, Yoonhee Kim, Aurelio Tobias, Chris Fook Sheng Ng, Xerxes Seposo, Yuming Guo, Yasushi Honda, Antonio Gasparrini, Ben Armstrong, Masahiro Hashizume

Table of Contents

[Figure S1. Flowchart illustrating the main stages of the statistical analysis 2](#_Toc44486832)

[Statistical analysis for Stage I: Seasonality assessment 3](#_Toc44486833)

[Table S1. The pooled peak-to-trough ratio (95% confidence intervals) for Japan as a whole for all-cause mortality by using different degrees of freedom $\boldsymbol{(df}\boldsymbol{)}$ for cyclic spline^*^ and natural cubic spline^§^ 5](#_Toc44486834)

[Table S2. The 44-year averaged prefecture-specific peak-to-trough ratios (95% confidence intervals) for all-cause, circulatory and respiratory mortality 6](#_Toc44486835)

[Table S3. The relationship (slope estimate (95% confidence intervals)) between each prefecture-specific meta-predictor and PTR before adjusting for other meta-predictors 8](#_Toc44486836)

[Table S4. The relationship (slope estimate (95% confidence intervals)) between each prefecture-specific meta-predictor and PTR after adjusting for all the other meta-predictors 9](#_Toc44486837)

[Figure S3. Monthly mean of daily mean temperature and daily mortality cases at national level between 1972 and 2015 10](#_Toc44486838)

[Figure S4. Daily mean temperature and daily mortality cases from 1972 to 2015 at national level 11](#_Toc44486839)

[The spatial distribution of averaged daily mean temperature and mortality cases 12](#_Toc44486840)

[Spatial distribution of PTR before and after temperature adjustment by using the data for the overall study period of 44 years 13](#_Toc44486841)

[Figure S7. Time-series scatter plot for annual data on each prefecture-specific characteristic 14](#_Toc44486842)

Daily mortality

Time series regression analysis

Daily mean temperature

Temperature unadjusted

*(using data in each single year)*

Temperature adjusted

*(using 44 years of data)*

Temperature unadjusted

*(using 44 years of data)*

Using the number of daily deaths estimated to obtain yearly PTR for each single year

(Result: Figure 2 and 3)

Using estimated number of daily deaths to obtain peak-to-trough ratio (PTR)

Using estimated number of daily deaths from models with temperature adjustment, that is once those deaths related with temperature were excluded, to obtain adjusted PTR

**Stage II**

**Stage I**

Annual mean temperature and other prefecture-specific characteristics

Compare temperature-adjusted PTR with unadjusted PTR to quantify the contribution of temperature to seasonality of mortality

(Result: Figure 1)

Multilevel multivariate meta-regression model

Evaluating the association between annual mean temperature and yearly PTR

(Result: Table 2)

Hypothesis 1: Seasonality of mortality is substantially driven by temperature

Hypothesis 2: Seasonal amplitude is decreasing under a warming climate

# Figure S1. Flowchart illustrating the main stages of the statistical analysis

# Statistical analysis for Stage I: Seasonality assessment

*(complementary to the description in main text):*

The seasonality of mortality was estimated using time series regression analysis (without and with temperature adjustment), assumed Poisson variation and allowed for overdispersion:

$$Temperatrue \boldsymbol{unadjusted}:$$

$Log\left( Y_{t} \right)=\alpha+cs\left( doy,4 \right)+ns\left( {influenza}_{t},3 \right)+\lambda{Strata}_{t}$

$$Temperature \boldsymbol{adjusted}:$$

$$Log\left( Y_{t} \right)=\alpha+cs\left( doy,4 \right)+ns\left( {influenza}_{t},3 \right)+\lambda{Strata}_{t}+\beta{Temp}_{t,l}$$

*t*: the day of the observation;

$Y_{t}$: the observed daily numbers of mortality on day *t*;

$\alpha$: the intercept;

*doy*: day of year (for non-leap years, values were taken from 1 to 366; for leap years, values were taken from 1 to 59 from the 1st day to the 59th day (i.e., February 28th) and 61 to 366 from the 60th day to the 365th day in common years), which was fitted using cyclic cubic spline with 4 degrees of freedom (*df*);

${influenza}_{t}$: the observed daily numbers of mortality due to influenza on day t, which was controlled using natural cubic spline with 3 *df*;

${Strata}_{t}$: strata defined by year, day of week, and their interaction to control for the long-term trend and the effect of day of week, and $\lambda$ is the vector of coefficients;

${Temp}_{t,l}$: a matrix obtained by using cross basis function to temperature; *l* is the lag days, and $\beta$ is the vector of coefficients. (For the cross basis function, a natural cubic B-spline basis with three internal knots at the 25th, 50th, and 75th percentiles of temperature distribution was used for exposure-response association, and another natural cubic B-spline basis with 3 *df* with extended lag up to 21 days was used for the lag-response association.)

In addition, two large earthquakes that caused many deaths in Japan,^1,2^ the Great Hanshin earthquake (5,488 deaths; 17 January 1995) and Great East Japan earthquake (15,782 deaths; 11 March 2011), were controlled for by incorporating a binary variable in the model assigning 1 to each day in January 1995 and March 2011 for the affected prefectures and 0 to the other days. Both earthquakes occurred in the cold season, which, if not considered, would increase the mortality peaks for those years. The prefectures affected were identified from meteorological records.^3^

Figure S2. Daily numbers of observed circulatory mortalities in Japan between 1972 and 2015 and the estimated numbers of daily circulatory mortality from time series regression models

Grey dot: daily numbers of observed mortality cases in Japan between 1972 and 2015;

Blue: pooled mortality estimates with 95% confidence intervals obtained from prefecture-specific estimates from models without temperature adjustment

Red: pooled mortality estimates with 95% confidence intervals obtained from prefecture-specific estimates from models with temperature adjustment, i.e., removed the short-term effect of temperature on mortality

Figure S2 shows daily numbers of observed circulatory mortalities in Japan between 1972 and 2015 and estimated daily circulatory mortality between 1972 and 2015. The days with the maximum and minimum estimated mortality were identified as peaks and troughs, respectively. The peak-to-trough ratio (PTR) of mortality estimates was then obtained as a measure of seasonal amplitude. We then compared PTR before and after adjusting for temperature to quantify the contribution of temperature to seasonality of mortality.

Reference:

1. Osamu, K., Masumi, A. & Etsuko, K. The medical and public health response to the Great Hanshin-Awaji Earthquake in Japan : a case study in disaster planning. *Med. Glob. Surviv.* **2**, 214–226 (1995).

2. Kazama, M. & Noda, T. Damage statistics (Summary of the 2011 off the Pacific Coast of Tohoku Earthquake damage). *Soils Found.* **52**, 780–792 (2012).

3. Japan Meteorological Agency. Japan Meteorological Agency database (in Japanese) of seismic intensity. Available at: http://www.data.jma.go.jp/svd/eqdb/data/shindo/. (Accessed: 19th June 2019)

4. Japan Meteorological Agency. General Information on Climate of Japan. Available at: https://www.data.jma.go.jp/gmd/cpd/longfcst/en/tourist.html. (Accessed: 30th June 2019)

5. Medina-Ramón, M. & Schwartz, J. Temperature, temperature extremes, and mortality: a study of acclimatisation and effect modification in 50 US cities. *Occup. Environ. Med.* **64**, 827–33 (2007).

6. Braga, A. L. F., Zanobetti, A. & Schwartz, J. The effect of weather on respiratory and cardiovascular deaths in 12 U.S. cities. *Environ. Health Perspect.* **110**, 859–63 (2002).

7. Marti-Soler, H. *et al.* Seasonal Variation of Overall and Cardiovascular Mortality: A Study in 19 Countries from Different Geographic Locations. *PLoS One* **9**, e113500 (2014).

8. Stewart, S., Keates, A. K., Redfern, A. & McMurray, J. J. V. Seasonal variations in cardiovascular disease. *Nat. Rev. Cardiol.* **14**, 654–664 (2017).

| Table S1. The pooled peak-to-trough ratio (95% confidence intervals) for Japan as a whole for all-cause mortality by using different degrees of freedom $\boldsymbol{(df}\boldsymbol{)}$ for cyclic spline^*^ and natural cubic spline^§^ | | |
| --- | --- | --- |
|  | Temperature unadjusted | Temperature adjusted |
| Cyclic spline |  |  |
| 4$df$ (main models) | 1.284  (1.270,1.298) | 1.084  (1.077, 1.091) |
| 5$df$ | 1.293  (1.278, 1.307) | 1.083  (1.076, 1.091) |
| 6$df$ | 1.282  (1.268, 1.296) | 1.077  (1.069, 1.086) |
| Natural cubic spline |  |  |
| 2$df$ | 1.284  (1.270,1.298) | 1.084  (1.077, 1.091) |
| 3$df$(main models) | 1.285  (1.271, 1.300) | 1.081  (1.074, 1.088) |
| *^*^Cyclic spline function for day of year*  *^§^Natural cubic spline function for influenza adjustment* | | |

| **Table S2**. The 44-year averaged prefecture-specific peak-to-trough ratios (95% confidence intervals) for all-cause, circulatory and respiratory mortality | | | | | | |
| --- | --- | --- | --- | --- | --- | --- |
| Prefectures | All-cause mortality | | Circulatory mortality | | Respiratory mortality | |
|  | Temperature Unadjusted | Temperature Adjusted | Temperature Unadjusted | Temperature Adjusted | Temperature Unadjusted | Temperature Adjusted |
| Aichi | 1.280  (1.274-1.286) | 1.116  (1.082-1.151) | 1.529  (1.516-1.542) | 1.153  (1.093-1.216) | 1.459  (1.437-1.482) | 1.37  (1.235-1.519) |
| Akita | 1.223  (1.212-1.234) | 1.084  (1.032-1.138) | 1.428  (1.407-1.449) | 1.163  (1.057-1.278) | 1.367  (1.333-1.402) | 1.562  (1.278-1.909) |
| Aomori | 1.188  (1.177-1.198) | 1.077  (1.029-1.128) | 1.379  (1.359-1.4) | 1.135  (1.068-1.207) | 1.342  (1.308-1.376) | 1.448  (1.211-1.731) |
| Chiba | 1.292  (1.285-1.298) | 1.098  (1.066-1.131) | 1.538  (1.524-1.552) | 1.087  (1.065-1.109) | 1.448  (1.426-1.471) | 1.434  (1.291-1.591) |
| Ehime | 1.312  (1.302-1.322) | 1.113  (1.069-1.16) | 1.584  (1.563-1.606) | 1.08  (1.01-1.155) | 1.406  (1.374-1.438) | 1.457  (1.221-1.74) |
| Fukui | 1.303  (1.289-1.318) | 1.091  (1.038-1.147) | 1.555  (1.526-1.584) | 1.146  (1.04-1.261) | 1.42  (1.377-1.463) | 1.233  (1.024-1.485) |
| Fukuoka | 1.273  (1.266-1.28) | 1.075  (1.041-1.109) | 1.491  (1.477-1.505) | 1.099  (1.047-1.154) | 1.476  (1.455-1.497) | 1.416  (1.277-1.57) |
| Fukushima | 1.326  (1.316-1.335) | 1.134  (1.086-1.185) | 1.588  (1.569-1.607) | 1.165  (1.089-1.246) | 1.509  (1.479-1.54) | 1.359  (1.18-1.565) |
| Gifu | 1.312  (1.302-1.322) | 1.106  (1.067-1.148) | 1.589  (1.569-1.61) | 1.194  (1.104-1.291) | 1.466  (1.435-1.498) | 1.287  (1.093-1.516) |
| Gunma | 1.314  (1.304-1.324) | 1.092  (1.054-1.132) | 1.541  (1.522-1.56) | 1.109  (1.042-1.18) | 1.477  (1.445-1.51) | 1.576  (1.357-1.831) |
| Hiroshima | 1.288  (1.28-1.297) | 1.066  (1.028-1.106) | 1.558  (1.54-1.576) | 1.065  (1.004-1.129) | 1.463  (1.437-1.49) | 1.485  (1.29-1.709) |
| Hokkaido | 1.116  (1.11-1.122) | 1.093  (1.058-1.13) | 1.269  (1.258-1.28) | 1.157  (1.095-1.222) | 1.222  (1.205-1.24) | 1.285  (1.165-1.417) |
| Hyogo | 1.28  (1.274-1.287) | 1.1  (1.062-1.139) | 1.536  (1.522-1.55) | 1.068  (1.054-1.083) | 1.47  (1.449-1.492) | 1.422  (1.275-1.586) |
| Ibaraki | 1.331  (1.323-1.34) | 1.108  (1.065-1.152) | 1.597  (1.579-1.615) | 1.12  (1.049-1.196) | 1.593  (1.56-1.626) | 1.425  (1.241-1.636) |
| Ishikawa | 1.257  (1.245-1.268) | 1.076  (1.03-1.123) | 1.481  (1.457-1.506) | 1.136  (1.037-1.245) | 1.384  (1.349-1.42) | 1.383  (1.122-1.705) |
| Iwate | 1.271  (1.26-1.281) | 1.079  (1.033-1.127) | 1.496  (1.476-1.517) | 1.066*****  (0.991-1.146) | 1.482  (1.448-1.517) | 1.619  (1.359-1.928) |
| Kagawa | 1.329  (1.316-1.342) | 1.05  (1.034-1.067) | 1.611  (1.583-1.639) | 1.077  (0.963-1.204) | 1.512  (1.473-1.553) | 1.196  (1.022-1.401) |
| Kagoshima | 1.334  (1.324-1.345) | 1.065  (1.028-1.103) | 1.563  (1.544-1.583) | 1.067  (1.01-1.128) | 1.595  (1.562-1.629) | 1.351  (1.203-1.516) |
| Kanagawa | 1.25  (1.244-1.256) | 1.072  (1.044-1.101) | 1.438  (1.426-1.45) | 1.092  (1.044-1.143) | 1.423  (1.401-1.444) | 1.325  (1.208-1.452) |
| Kochi | 1.31  (1.297-1.323) | 1.064  (1.008-1.122) | 1.517  (1.492-1.541) | 1.126  (1.027-1.235) | 1.513  (1.47-1.557) | 1.231  (1.023-1.481) |
| Kumamoto | 1.326  (1.317-1.336) | 1.074  (1.038-1.112) | 1.573  (1.553-1.593) | 1.111  (1.044-1.181) | 1.546  (1.514-1.578) | 1.205  (1.055-1.375) |
| Kyoto | 1.266  (1.257-1.275) | 1.067  (1.03-1.105) | 1.531  (1.512-1.549) | 1.069  (1.028-1.111) | 1.407  (1.379-1.435) | 1.275  (1.111-1.463) |
| Mie | 1.332  (1.322-1.342) | 1.119  (1.067-1.174) | 1.588  (1.567-1.609) | 1.109  (1.037-1.185) | 1.497  (1.463-1.532) | 1.537  (1.279-1.847) |
| Miyagi | 1.293  (1.284-1.303) | 1.117  (1.078-1.157) | 1.55  (1.531-1.569) | 1.134  (1.078-1.192) | 1.475  (1.443-1.508) | 1.491  (1.278-1.74) |
| Miyazaki | 1.324  (1.312-1.336) | 1.053  (1.012-1.096) | 1.584  (1.559-1.61) | 1.075  (0.973-1.188) | 1.563  (1.522-1.605) | 1.265  (1.072-1.494) |
| Nagano | 1.323  (1.313-1.332) | 1.078  (1.042-1.115) | 1.566  (1.548-1.584) | 1.067  (1.011-1.126) | 1.502  (1.472-1.532) | 1.184  (1.033-1.357) |
| Nagasaki | 1.282  (1.272-1.293) | 1.069  (1.019-1.122) | 1.506  (1.485-1.527) | 1.085  (1.022-1.153) | 1.491  (1.46-1.524) | 1.493  (1.269-1.755) |
| Nara | 1.294  (1.282-1.305) | 1.066  (1.015-1.118) | 1.545  (1.52-1.571) | 1.038  (1.013-1.064) | 1.443  (1.406-1.481) | 1.285  (1.056-1.564) |
|  |  |  |  |  |  |  |
|  |  |  |  |  |  |  |
| **Table S2**. Continued. | | | | | | |
| Prefectures | All-cause mortality | | Circulatory mortality | | Respiratory mortality | |
|  | Temperature Unadjusted | Temperature Adjusted | Temperature Unadjusted | Temperature Adjusted | Temperature Unadjusted | Temperature Adjusted |
| Niigata | 1.265  (1.256-1.273) | 1.138  (1.098-1.179) | 1.514  (1.497-1.53) | 1.231  (1.156-1.31) | 1.364  (1.339-1.389) | 1.486  (1.283-1.721) |
| Oita | 1.292  (1.28-1.303) | 1.076  (1.016-1.139) | 1.551  (1.528-1.575) | 1.041  (0.956-1.133) | 1.495  (1.456-1.535) | 1.313  (1.091-1.581) |
| Okayama | 1.318  (1.308-1.327) | 1.074  (1.037-1.112) | 1.594  (1.574-1.615) | 1.094  (1.022-1.172) | 1.453  (1.425-1.482) | 1.17  (1.036-1.321) |
| Okinawa | 1.248  (1.233-1.264) | 1.106  (1.057-1.158) | 1.383  (1.352-1.416) | 1.064*****  (0.993-1.139) | 1.63  (1.579-1.683) | 1.504  (1.323-1.71) |
| Osaka | 1.228  (1.222-1.233) | 1.087  (1.054-1.121) | 1.442  (1.431-1.454) | 1.068  (1.015-1.122) | 1.391  (1.373-1.41) | 1.233  (1.125-1.351) |
| Saga | 1.321  (1.308-1.335) | 1.157  (1.094-1.223) | 1.573  (1.544-1.602) | 1.132  (1.036-1.237) | 1.558  (1.515-1.603) | 1.523  (1.233-1.881) |
| Saitama | 1.278  (1.271-1.285) | 1.08  (1.047-1.113) | 1.517  (1.503-1.531) | 1.072  (1.057-1.087) | 1.458  (1.434-1.482) | 1.443  (1.297-1.605) |
| Shiga | 1.316  (1.303-1.329) | 1.079  (1.028-1.132) | 1.611  (1.584-1.638) | 1.11  (1.015-1.213) | 1.517  (1.473-1.562) | 1.48  (1.188-1.844) |
| Shimane | 1.314  (1.3-1.327) | 1.052  (1-1.107) | 1.596  (1.567-1.625) | 1.236  (1.094-1.398) | 1.516  (1.472-1.56) | 1.189  (0.963-1.468) |
| Shizuoka | 1.333  (1.325-1.34) | 1.074  (1.041-1.107) | 1.597  (1.581-1.614) | 1.114  (1.056-1.175) | 1.537  (1.509-1.566) | 1.318  (1.16-1.497) |
| Tochigi | 1.34  (1.331-1.35) | 1.127  (1.073-1.184) | 1.607  (1.586-1.627) | 1.166  (1.079-1.26) | 1.574  (1.538-1.61) | 1.454  (1.243-1.699) |
| Tokushima | 1.306  (1.293-1.319) | 1.095  (1.026-1.168) | 1.555  (1.527-1.583) | 1.164  (1.038-1.305) | 1.476  (1.436-1.518) | 1.409  (1.121-1.771) |
| Tokyo | 1.234  (1.229-1.239) | 1.116  (1.089-1.143) | 1.428  (1.418-1.438) | 1.103  (1.061-1.147) | 1.381  (1.364-1.398) | 1.461  (1.358-1.571) |
| Tottori | 1.322  (1.306-1.338) | 1.083  (1.013-1.157) | 1.606  (1.573-1.639) | 1.172  (1.064-1.293) | 1.545  (1.49-1.603) | 1.335  (1.032-1.729) |
| Toyama | 1.242  (1.23-1.254) | 1.092  (1.035-1.152) | 1.449  (1.426-1.474) | 1.066  (0.971-1.17) | 1.355  (1.321-1.389) | 1.26  (1.038-1.53) |
| Wakayama | 1.3  (1.288-1.312) | 1.064  (1.024-1.105) | 1.545  (1.521-1.568) | 1.084  (1.016-1.157) | 1.432  (1.393-1.472) | 1.178  (0.953-1.456) |
| Yamagata | 1.271  (1.259-1.282) | 1.092  (1.041-1.146) | 1.492  (1.471-1.514) | 1.119  (1.027-1.219) | 1.425  (1.39-1.461) | 1.419  (1.197-1.681) |
| Yamaguchi | 1.277  (1.268-1.287) | 1.067  (1.019-1.117) | 1.52  (1.499-1.54) | 1.071  (0.995-1.153) | 1.45  (1.419-1.482) | 1.371  (1.165-1.614) |
| Yamanashi | 1.355  (1.34-1.369) | 1.056  (0.976-1.143) | 1.634  (1.603-1.665) | 1.028  (0.914-1.156) | 1.601  (1.551-1.653) | 1.555  (1.226-1.972) |
| **Pooled** | **1.284**  **(1.270-1.298)** | **1.084**  **(1.077-1.091)** | **1.527**  **(1.504-1.549)** | **1.096**  **(1.082-1.110)** | **1.459**  **(1.437-1.481)** | **1.354**  **(1.323-1.387)** |

| **Table S3.** The relationship (slope estimate (95% confidence intervals)) between each prefecture-specific meta-predictor and PTR before adjusting for other meta-predictors | | | |
| --- | --- | --- | --- |
| **Meta predictors** | **All-cause mortality** | **Circulatory mortality** | **Respiratory mortality** |
| **Climate** | | | |
| Tmean (℃) | -0.016  (-0.021, -0.012) | -0.021  (-0.027, -0.015) | -0.023  (-0.034, -0.010) |
| RHmean (%) | 0.003  (0.002, 0.004) | 0.003  (0.002, 0.005) | 0.009  (0.006, 0.012) |
| **Demographic, age (%)** |  |  |  |
| ≥65 y (%) | 0.623  (0.394, 0.853) | 0.197  (-0.131, 0.525) | 1.702  (1.102, 2.302) |
| **Macroeconomics** |  |  |  |
| CPI | 0.001  (0.0003, 0.002) | 0.002  (0.001, 0.002) | -0.002  (-0.005, 0.0001) |
| **Air conditioning (%)** |  |  |  |
| AC (%) | -0.0002  (-0.001, 0.0001) | -0.0002  (-0.0005, 0.0002) | -0.0012  (-0.002, -0.0003) |
| Tmean: mean temperature; RHmean: mean relative humidity; ≥65 y: proportion of individuals ≥65 years old;  CPI: Consumer Price Index; AC: the prevalence of air conditioning; | | | |

| **Table S4.** The relationship (slope estimate (95% confidence intervals)) between each prefecture-specific meta-predictor and PTR after adjusting for all the other meta-predictors | | | |
| --- | --- | --- | --- |
| **Meta predictors** | **All-cause mortality** | **Circulatory mortality** | **Respiratory mortality** |
| **Climate** | | | |
| Tmean (℃) | -0.010  (-0.014, -0.005) | -0.014  (-0.020, -0.008) | -0.001  (-0.015, 0.012) |
| RHmean (%) | 0.002  (0.001, 0.003) | 0.003  (0.001, 0.004) | 0.009  (0.006, 0.012) |
| **Demographic, age (%)** |  |  |  |
| ≥65 y (%) | 0.547  (0.308, 0.786) | 0.049  (-0.300, 0.394) | 1.098  (0.491, 1.704) |
| **Macroeconomics** |  |  |  |
| CPI | 0.002  (0.001, 0.002) | 0.001  (0.0004, 0.002) | 0.002  (-0.001, 0.004) |
| **Air conditioning (%)** |  |  |  |
| AC (%) | -0.0001  (-0.0004, 0.0002) | -0.0001  (-0.0005, 0.0002) | -0.0007  (-0.0020, 0.0001) |
| Tmean: mean temperature; RHmean: mean relative humidity; ≥65 y: proportion of individuals over 65 years old;  CPI: Consumer Price Index; AC: the prevalence of air conditioning;  *$p<0.05$; | | | |

# **Figure S3.** Monthly summary of temperature and mortality cases at national level between 1972 and 2015

# **Figure S4.** Daily mean temperature and daily mortality cases from 1972 to 2015 at national level

*(Data on 1995/01/17 and 2011/03/11 was removed in the figure, due to the extremely large amount of all-cause mortality cases (8490 and 21, 531 cases, respectively) caused by two big earthquakes on these two days)*

# The spatial distribution of averaged daily mean temperature and mortality cases

**Figure S5.** Averaged daily mean temperature (orange) and averaged daily mortality cases between 1972 and 2015 in each prefecture for all-cause, circulatory and respiratory mortality (from left to right)

Figure S5 shows the spatial distribution of averaged daily mean temperature and daily mortality cases. In general, eastern Japan had a lower temperature than western Japan. Hokkaido had the lowest temperatures and highest mortality.

# Spatial distribution of PTR before and after temperature adjustment by using the data for the overall study period of 44 years

**Figure S6.** Prefecture-specific peak-to-trough ratios (PTR) for all-cause (top, green), circulatory (middle, red) and respiratory (bottom, blue) mortality (left: temperature unadjusted; right: temperature adjusted) by using 44 years of data

Figure S6 shows the spatial variation of prefecture-specific PTRs before and after temperature adjustment. Prior to temperature adjustment, PTRs were generally lower in some eastern provinces on the Sea of Japan (East Sea), with the lowest PTR observed in Hokkaido. These prefectures generally experience very cold winters with heavy snow and warm summers (Japan Meteorological Agency). Studies conducted in multiple cities and countries found that regions with cold winters exhibited less distinct seasonality of mortality than regions with moderate climates.^5–7^ Adaptation to colder regions, which includes habit change, metabolic adjustment, and insulative acclimatization, may contribute to lower circulatory-related seasonality.^8^

Reference:

1. Osamu, K., Masumi, A. & Etsuko, K. The medical and public health response to the Great Hanshin-Awaji Earthquake in Japan : a case study in disaster planning. *Med. Glob. Surviv.* **2**, 214–226 (1995).

2. Kazama, M. & Noda, T. Damage statistics (Summary of the 2011 off the Pacific Coast of Tohoku Earthquake damage). *Soils Found.* **52**, 780–792 (2012).

3. Japan Meteorological Agency. Japan Meteorological Agency database (in Japanese) of seismic intensity. Available at: http://www.data.jma.go.jp/svd/eqdb/data/shindo/. (Accessed: 19th June 2019)

4. Japan Meteorological Agency. General Information on Climate of Japan. Available at: https://www.data.jma.go.jp/gmd/cpd/longfcst/en/tourist.html. (Accessed: 30th June 2019)

5. Medina-Ramón, M. & Schwartz, J. Temperature, temperature extremes, and mortality: a study of acclimatisation and effect modification in 50 US cities. *Occup. Environ. Med.* **64**, 827–33 (2007).

6. Braga, A. L. F., Zanobetti, A. & Schwartz, J. The effect of weather on respiratory and cardiovascular deaths in 12 U.S. cities. *Environ. Health Perspect.* **110**, 859–63 (2002).

7. Marti-Soler, H. *et al.* Seasonal Variation of Overall and Cardiovascular Mortality: A Study in 19 Countries from Different Geographic Locations. *PLoS One* **9**, e113500 (2014).

8. Stewart, S., Keates, A. K., Redfern, A. & McMurray, J. J. V. Seasonal variations in cardiovascular disease. *Nat. Rev. Cardiol.* **14**, 654–664 (2017).

# **Figure S7.** Time-series scatter plot for annual data on each prefecture-specific characteristic
